# Senescence-Related lncRNA Signature Predicts Prognosis, Response to Immunotherapy and Chemotherapy in Skin Cutaneous Melanoma

**DOI:** 10.3390/biom13040661

**Published:** 2023-04-09

**Authors:** Kefan Lin, Yingtong Zhou, Yanling Lin, Yuanyuan Feng, Yuting Chen, Longmei Cai

**Affiliations:** 1 Department of Radiation Oncology, Nanfang Hospital, Southern Medical University, Guangzhou 510515, China; linkefan316@outlook.com (K.L.); lystone3@outlook.com (Y.Z.); 1120011070@smu.edu.cn (Y.L.); fyy2333@outlook.com (Y.F.); 2 First Clinical Medical College, Southern Medical University, Guangzhou 510515, China; yutingcc2023@outlook.com

**Keywords:** senescence, lncRNA, melanoma, prognostic signature, tumor immune microenvironment, immunotherapy, tumor burden mutation, chemotherapy drugs

## Abstract

Skin cutaneous melanoma (SKCM) is a highly malignant and aggressive cancer. Previous studies have shown that cellular senescence is a promising therapeutic strategy to limit melanoma cell progression. However, models to predict the prognosis of melanoma based on senescence-related lncRNAs and the efficacy of immune checkpoint therapy remain undefined. In this study, we developed a predictive signature consisting of four senescence-related lncRNAs (AC009495.2, U62317.1, AATBC, MIR205HG), and we then classified patients into high- and low-risk groups. GSEA (Gene set enrichment analysis) showed different activation of immune-related pathways in two groups. In addition, there were significant differences between the scores of tumor immune microenvironment, tumor burden mutation, immune checkpoint expression, and chemotherapeutic drug sensitivity between the two groups of patients. It provides new insights to guide more personalized treatment for patients with SKCM.

## 1. Introduction

Skin cutaneous melanoma (SKCM) is a pernicious and aggressive cancer resulting from melanocytes and usually caused by sun exposure [1]. As is shown in epidemiological studies, an estimated 192,000 new diagnoses of SKCM are reported every year in the United States, six times more than 40 years ago [2]. Most newly diagnosed patients with melanoma are in the early stages. Under most circumstances, surgical excision remains a curative treatment option for these patients [3]. However, metastatic melanoma patients have a cure rate of only 23%. For these patients, molecularly targeted therapy and immune checkpoint therapy are the main treatment strategies [4]. Nevertheless, due to individual variability, different efficacy of drugs leads to different prognoses. Therefore, to evaluate the prognosis and personalize treatment for patients, it is necessary to develop new and effective markers.

Cellular senescence in cells is defined as a stalled state in the cell cycle triggered by multiple types of stress. Involved in cellular pathological and physiological processes, it is characterized by excessive secretion of complex pro-inflammatory secretory phenotypes (SASP) and can therefore interact with the tumor microenvironment on its way to influence tumor progression [5]. During the early tumorigenic stage, oncogenic signals are gradually enhanced until the threshold of oncogenic signals p16 and p53 are breached. Additionally, inhibitors of the cell cycle would block this signaling, allowing cancer cells to enter senescence, which prevents the expansion of cancer cells [6]. It is recognized that oncogene activation causes proliferative stress in mammalian cells; however, senescence induction contributes to the progression of tumor lesions from benign to malignant [7]. Therefore, induction of senescence production for the treatment of cancer is increasingly becoming a therapeutic option. Antitumor approaches, including radiotherapy and chemotherapy, are then able to induce the production of senescence by causing DNA damage [8].

An endogenous collection of RNAs greater than 200 base pairs in length, long non-coding RNAs (lncRNAs) play a diverse range of biological roles. According to previous studies, lncRNAs are versatile molecules involved in various tumorigenic processes and offer the possibility of being used as biomarkers [9,10]. In patients with colorectal cancer (CRC), stomach adenocarcinoma, or Lung adenocarcinoma (LUAD), senescence-related lncRNA models have been developed and have certain predictive values [11,12,13]. However, the signature of senescence-related lncRNAs has not yet been established and applied to the prognosis prediction and clinical treatment of SKCM patients.

In this research, one prognostic signature was established for senescence-associated lncRNAs that were differentially expressed in SKCM patients. Subsequently, for the prediction of overall survival in SKCM patients, we developed a nomogram. In addition to this, we investigated the relationship between risk score with tumor immune infiltration, immune checkpoints, and chemotherapeutic susceptibility of drugs. It is demonstrated that the signature can efficiently predict the prognosis of skin cutaneous melanoma patients, which can help guide clinical decisions and optimize therapeutic options.

## 2. Materials and Methods

### 2.1. Data Acquisition and Normalization

**Figure 1:**
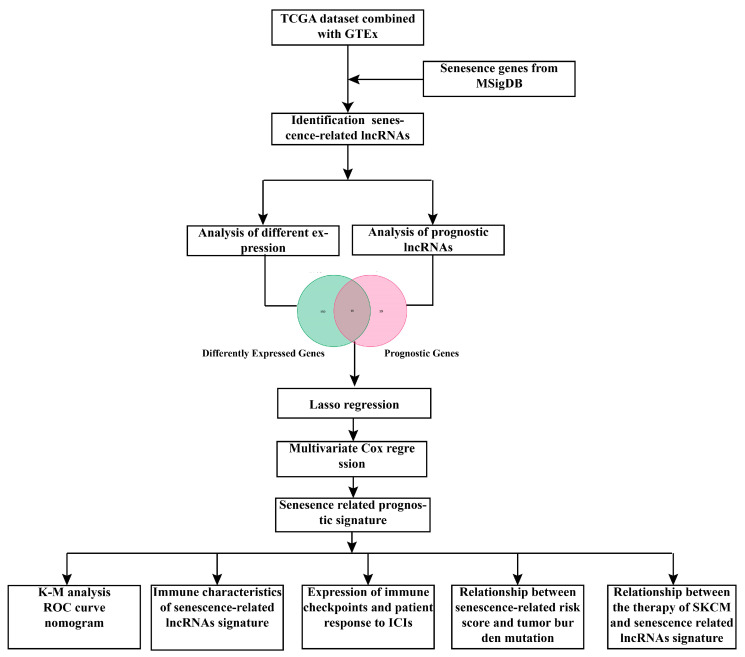
Process flowchart for analysis. TCGA, the Cancer Genome Atlas; GTEx: the Genotype Tissue Expression database. MSigDB: the Molecular Signatures Database. lncRNAs: long non-coding RNAs; K-M analysis: Kaplan–Meier approach for survival analysis. ROC curve: the receiver operating characteristic curve. ICIs: immune checkpoint inhibitors. SKCM: skin cutaneous melanoma.

The process flowchart for analysis is illustrated in Figure 1. A total of 447 SKCM patients were included in our study group after we excluded patients with a survival time of fewer than 30 days and with no survival time from The Cancer Genome Atlas (TCGA), which we obtained from the UCSC Xena (http://xena.ucsc.edu/, accessed on 6 June 2022). Next, the TCGA cohort was then randomly divided into two parts, training (50%) and test (50%) sets. In Supplementary Table S1, the clinical characteristics of the TCGA cohort in the different risk groups are summarized. In addition, we downloaded tissue samples from 813 normal individuals from the Genotype Tissue Expression database (https://gtexportal.org/home/, accessed on 10 June 2022) (FPKM (fragments per kilobase of transcript per million fragments mapped), log2(x + 0.001) transformed) as a normal control for the TCGA dataset. In Supplementary Table S2, senescence-related genes were downloaded from the MSigDB database [14].

### 2.2. Prognosis-Related lncRNAs and Differentially Expressed lncRNAs

Utilizing data from the TCGA cohort, 179 senescence-related lncRNAs were identified as differentially expressed in normal and tumor samples by “limma” package (version 3.50.3) (*p*-value < 0.05, log FC (fold change) > 0.586) [15]. Using the univariate Cox regression, we identified 48 senescence-related long noncoding RNAs implicated in prognosis. Candidate genes were found by intersecting different lncRNAs and prognosis-related lncRNAs, and their expression-related network was visualized by “graph” package (version 1.50.0, https://www.rdocumentation.org/packages/graph/versions/1.50.0, accessed on 20 July 2022).

### 2.3. Development of Senescence-Related lncRNAs Signature

Furthermore, to minimize overfitting effects and construct a better risk signature, Lasso regression and multivariate Cox regression were used to construct the prognostic signature, and finally, four lncRNAs were included. The calculation was as follows: $riskscore=\sum {Coef}_{lncRNAs}\times {Exp}_{lncRNAs}$, where ${Exp}_{lncRNAs}$ and ${Coef}_{lncRNAs}$ represented the expression and regression coefficient of signature lncRNAs. To better characterize the different patients, with the median risk score as the cutoff value, patients were divided into two groups: high- and low-risk. “RMS” package (version 6.3-0, https://rdocumentation.org/packages/rms/versions/6.3-0, accessed on 15 June 2022) was employed to construct the nomogram integrating risk scores and clinical characteristics. Clustering visualization was performed using principal component analysis (PCA) [16].

### 2.4. Gene Set Enrichment Analysis and Construction of ceRNA Network

To further explore the biological characteristics of the two risk groups, gene set enrichment analysis (GSEA) was employed. *p*-values below 0.05 were considered as significant gene enrichment. Mircode and ENCORI (Starbase V3.0, https://starbase.sysu.edu.cn/, accessed on 10 January 2023) databases were employed to predict the miRNAs interacting with the four lncRNAs in our signature, after which miRDB (https://mirdb.org/, accessed on 10 January 2023), miRTarBase (https://mirtarbase.cuhk.edu.cn/, accessed on 10 January 2023), and TargetScan (https://www.targetscan.org/vert_80/, accessed on 10 January 2023) were utilized to identify the mRNAs interacting with the miRNAs, which were then intersected with the differentially expressed mRNAs across tumor and normal. Consequently, 65 mRNAs and 13 miRNAs that interacted with the four lncRNAs in the signature were obtained, and a ceRNA network was constructed through Cytoscape software (version 3.6.1, Paul Shannon, Seattle, Washington 98103, USA).

### 2.5. Assessment of Tumor Immune Microenvironment and Calculation of Tumor Burden Mutation

Immune cell infiltration files were extracted from Timer2.0 [17] containing seven immune infiltration algorithms, followed by bubble plots showing the relationship between the correlation coefficient and immune infiltration. Immune infiltration in SKCM was analyzed by “Cibersort” package (version v1.03) [18], and the expression of lncRNAs in the signature was correlated with the expression of immune cells by the Spearman correlation test. In addition, a comparison was also employed between different risk groups with “Estimate” package for stromal, immune, and estimation scores [19].

Utilizing the “maftools” R package (version 2.10.05), we analyzed the tumor burden mutation of senescence-related genes in both risk groups [20].

### 2.6. Predicting Patient Response to ICIs and the Assessment of Immune Checkpoint Molecules

Immune phenotype scores (IPS) obtained from https://tcia.at/ (accessed on 20 June 2022) were used to predict patient response to immune checkpoint inhibitors (ICIs). From previous studies, checkpoint molecules associated with ICI treatment responses were identified.

### 2.7. Prediction of Chemotherapy Drug Sensitivity

Different chemotherapeutic agents were tested with the “pRRophetic” package (version 0.5) to determine the IC50 (the half-maximal drug inhibitory concentration) of both risk groups. IC50 indicates the inhibitory effect of a substance on a specific biological or biochemical function.

### 2.8. Immunohistochemistry Staining

We obtained paraffin sections of 10 cases of melanoma and 5 cases of benign nevi tissue from Nanfang Hospital, Southern Medical University, Guangzhou, China, following approval by the Ethics Committee of the same institution. For immunohistochemical (IHC) staining, the primary antibodies used included PD-1 (Programmed Cell Death Ligand 1) (ab52587, IHC 1:200), CD11b (ab133357, IHC 1:200) purchased from Abcam (Cambridge, MA, USA), CD3 (17617-1-AP, IHC 1:100) and CD20 (60271-1-Ig, IHC 1:200) from Proteintech (Chicago, IL, USA). The sections were first baked at 60 °C for 30 min, followed by dewaxing and dehydration in xylene and ethanol. Then, antigen repair was conducted in a microwave oven using 3% hydrogen peroxide for 15 min to inactivate endogenous peroxidase. Subsequently, 5% bovine serum albumin (BSA) was applied for 30 min and incubated overnight at 4 °C with the diluted primary antibody. The next day, the slides were incubated for 1 h at room temperature with the secondary antibody, followed by DAB (diaminobenzidine) staining, hematoxylin staining, and ethanol dehydration. The images were then measured for Average Optical Density using ImageJ software (version 1.53c, Wayne Rasband, Bethesda, MD, USA). Finally, statistical analysis was carried out using Graphpad Prism9.0 (GraphPad Software Inc, San Diego, CA, USA).

### 2.9. Real-Time Quantitative PCR

Total RNA was extracted from A375, PIG1 cell lines, mouse tumor tissue, and skin tissue using TRIzol reagent, followed by cDNA synthesis using HiScript^®^ II QRT SuperMPlease (Vazyme, Nanjing, China) add developer and ix. qRT-PCR was conducted using SYBR^®^ Green Pro Taq HS Premix II (Accurate, Guangzhou, Guangdong). The CT values were standardized by GAPDH and 2(-ΔΔCT) values were calculated for relative quantification. Statistical analysis was performed using GraphPad Prism. The primers used are listed in the Supplementary Table S3.

### 2.10. Statistical Analysis

In this study, for all statistical analyses, the R-studio software (version 2022.02.1, https://posit.co/products/open-source/rstudio/, accessed on 10 March 2022) was employed. The Wilcoxon test was applied to assess differences between normal and tumor groups or between different risk groups. The predictive power of our established signatures was evaluated using ROC (the receiver operating characteristic) curves. Kaplan–Meier survival curves were employed to assess the differences between the survival times of patients in different risk groups.

## 3. Results

### 3.1. Identification of Differently Expressed lncRNAs and Prognosis-Related lncRNAs

**Figure 2:**
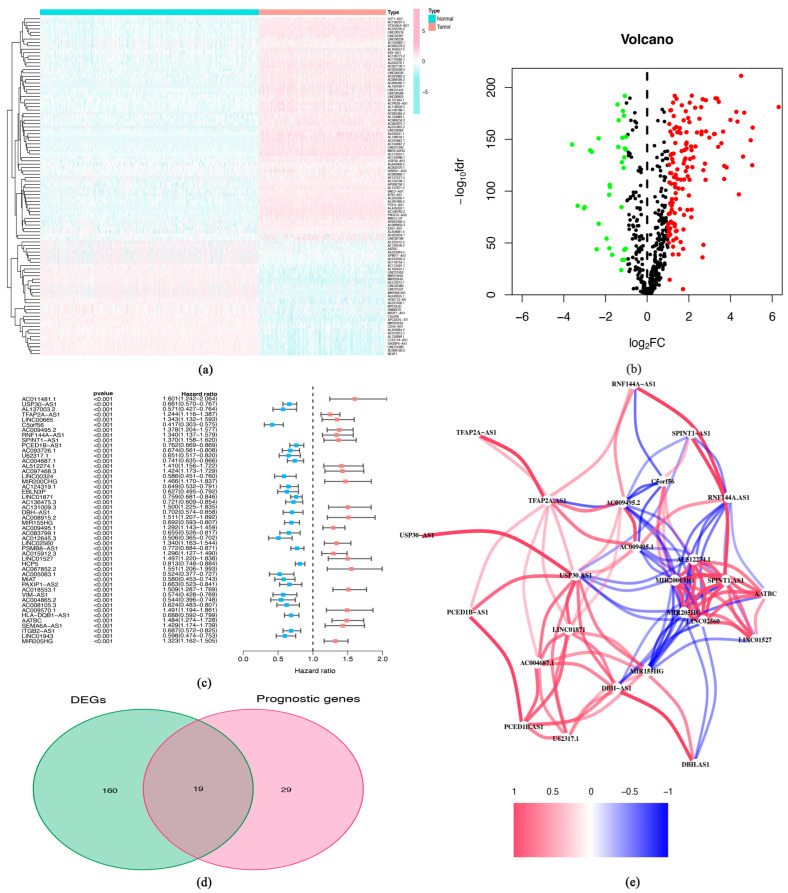
Identification of differently expressed lncRNAs and prognostic-related lncRNAs. (**a**,**b**) Heatmap and Volcano plots were used to demonstrate the differential expression of lncRNAs in normal and tumor tissues. In Volcano plots, the green plot represents down-regulated genes, the red plot represents up-regulated genes. (**c**) Univariate Cox regression was performed to identify forty-eight prognostic-related lncRNAs. (**d**) The intersection of differently expressed lncRNAs and prognostic-related lncRNAs. (**e**)The correlation network of intersected genes.

The expression of senescence-related lncRNAs was analyzed, and we identified the expression of 179 lncRNAs varied significantly between normal and tumor samples, with 145 genes being up-regulated and 34 genes down-regulated (Figure 2a,b). In addition, multivariate Cox regression was also applied to identify 48 lncRNAs related with prognosis (Figure 2c). By taking the intersection of the two groups of genes, we selected 19 lncRNAs for further analysis (Figure 2d). The expression-related networks of intersecting lncRNAs are depicted in Figure 2e.

### 3.2. Construction of Senescence-Related lncRNA Signature and Evaluation of Predictive Effects

**Figure 3:**
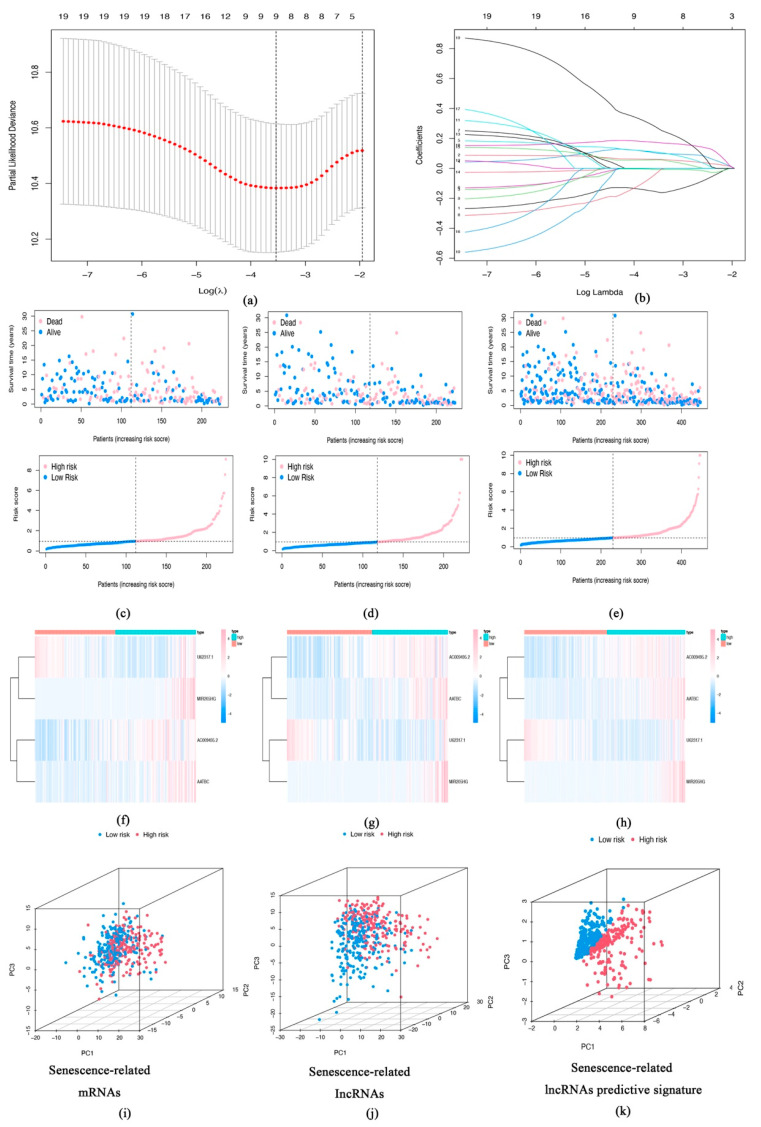
Construction of senescence-related lncRNA signature and evaluation of predictive effects. (**a**,**b**) Lasso regression analysis. (**c**–**e**) With increasing risk scores from left to right, the distribution of survival states was shown by the scatter plots in the training cohort (**c**), test cohort (**d**), and entire cohort (**e**). (**f**–**h**) The four senescence-related lncRNAs expression heatmap in training cohort (**f**), test cohort (**g**), and entire cohort (**h**). (**i**–**k**) Clustering visualization of patients in different risk groups was performed using principal component analysis (PCA). The blue plots represent low risk and the red plots represent high risk groups.

Lasso-Cox regression was executed with 1000 cross-validations to construct a risk profile for four lncRNAs (Figure 3a,b). Based on their expression levels and regression coefficients, risk scores were calculated for each patient. The risk score = expression (AC009495.2) × (0.232) + expression (U62317.1) × (−0.492) + expression (AATBC) × (0.270) + expression (MIR205HG) × (0.525). Patients were then classified into two groups: high risk and low risk. From left to right, the distribution of risk scores is exhibited by the scatter plot in increasing order (Figure 3c–e). Expression of senescence-related lncRNAs in both risk groups was demonstrated by heat map (Figure 3f–h). The expression of signature lncRNAs in the human melanoma cell line A375 and the human epidermal cell line HaCaT was verified in Supplementary Figure S1. The PCA with senescence-related mRNAs, senescence-related lncRNAs, and senescence-related lncRNA predictive signature were used to separate patients in TCGA cohort (Figure 3i–k). As seen in our study, the senescence-related lncRNA predictive signature could clearly distinguish different risk groups, proving the model’s validity.

**Figure 4:**
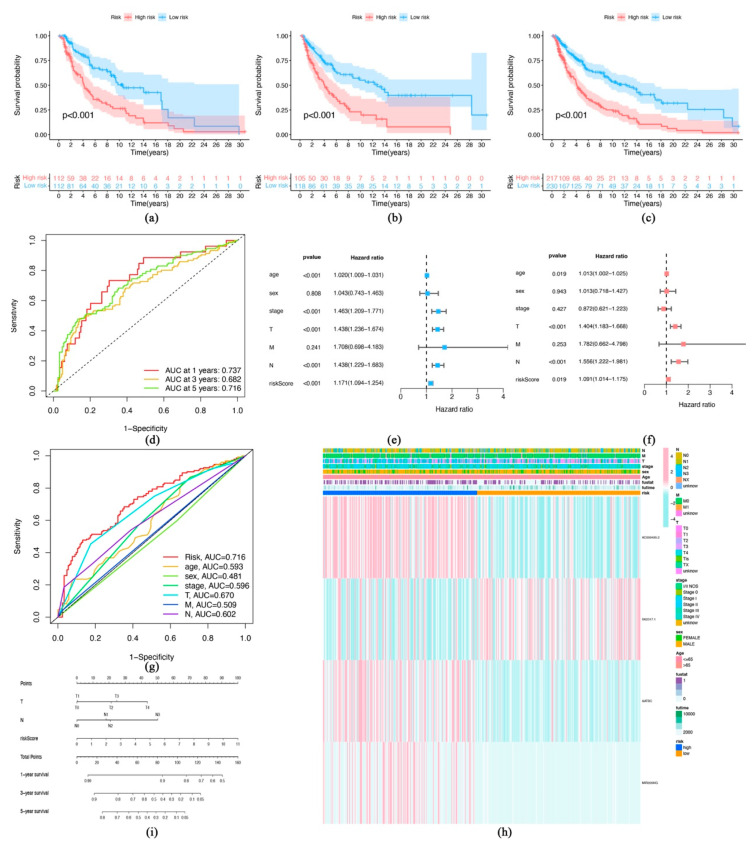
Verifying the accuracy of signatures. (**a**–**c**) Kaplan–Meier curves analysis of patients in both risk groups for the training cohort (**a**), test cohort (**b**), and entire cohort (**c**). (**d**) ROC curves showing AUCs for 1, 3, and 5 years in entire cohort. (**e**,**f**) Risk score and clinical characteristics of patients were analyzed using univariate Cox regression analysis (**e**) and multivariate Cox regression analysis (**f**). (**g**) ROC curves of clinical characteristics and risk score. (**h**) Heatmap of clinical characteristics and risk score. (**i**) The nomogram was constructed based on T, N stage, and risk score.

High-risk patients had shorter survival times than low-risk patients, according to K-M survival curves (Figure 4a–c). ROC curves showing AUCs for 1, 3, and 5 years were 0.737, 0.682, and 0.716 (Figure 4d), respectively. Through univariate Cox regression analysis, we found that risk score, T-stage, and N-stage have an impact on survival prognosis (*p* < 0.001) (Figure 4e). Then, again by multivariate Cox regression analysis, we identified risk score, T-stage, and N-stage were independent predictors of SKCM prognosis (Figure 4f). Additionally, the risk score showed the better prediction accuracy than T- and N-stage (Figure 4g). A heatmap of risk score and clinical characteristics are shown in Figure 4h. As shown in Figure 4i, we can predict the rate of survival of SKCM patients at different times by nomogram.

### 3.3. Immune Characteristics of Senescence-Related lncRNA Signature

**Figure 5:**
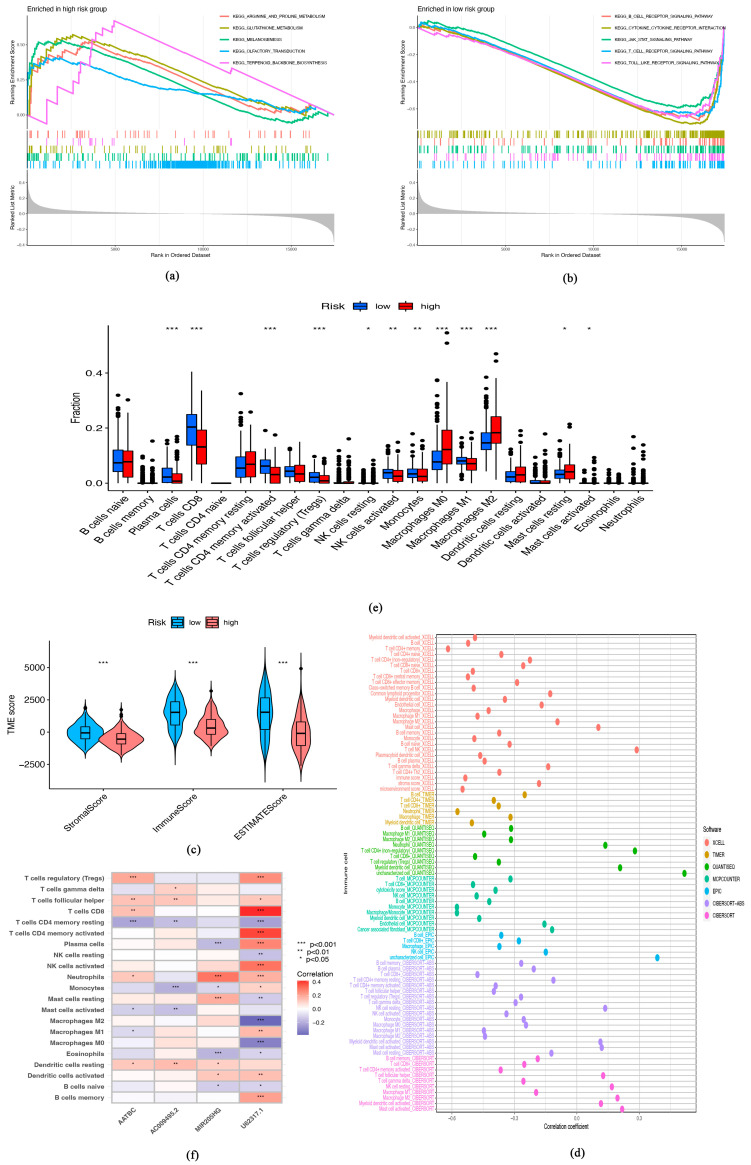
Immune characteristics of senescence-related lncRNA signature. (**a**,**b**) Gene set enrichment analysis between both risk groups. (**c**) Violin plot that demonstrated immune scores and stromal scores and that estimated scores for different risk groups in the Cancer Genoma Atlas (TCGA) cohort. (**d**) The infiltration of immune cells was calculated by seven immune algorithms. (**e**) The results calculated by the “Cibersort” algorithm. (**f**) The correlation between immune cells and the expression of signature lncRNA. * *p* < 0.05; ** *p* < 0.01; *** *p* < 0.001.

An increasing body of evidence has demonstrated that immune-related pathways play a key role in the development and progression of cancer. Over-activation of the JAK signaling pathway [21] and dysregulation in the BCR or TCR signaling pathways are closely associated with tumor growth [22,23]. To better understand the pathway enrichment, we performed GSEA in different risk groups. GSEA revealed that in the low-risk group, a majority of genes were enriched in immune-related pathways, including TOLL_LIKE_RECEPTOR_SIGNALING, JAK_STAT_SIGNALING, B_CELL_RECEPTOR_SIGNALING, CYTOKINE_CYTOKINE_RECEP TOR_INTERACTION, T_CELL_RECEP TOR_SIGNALING pathway (Figure 5a,b). Based on the results of the “Estimate” package, more immune cells and stromal cells were found to infiltrate in the low-risk group (Figure 5c). We also used seven immune algorithms including “QUANTISEQ, EPIC, MCPCOUNTER, Timer, Xcell, CIBERSORT, and CIBERSORT-ABS” to calculate the immune cell infiltration, and the correlation coefficient between risk score and immune cells was calculated using Spearman’s analysis, which demonstrated that the higher the risk score, the lower the infiltration of immune cells (Figure 5d). Through the “Cibersort” algorithm, higher expression levels of plasma cells, activated memory T cells, and CD8+ T cells were found in the low-risk group, whereas higher expression levels of M2 macrophages were found in the high-risk group (Figure 5e). In addition, the correlation between immune cells and the expression of lncRNA in the signature was also demonstrated in Figure 5f. We employed B16 melanoma cells to construct a subcutaneous tumor model in mice, and then evaluated the expression of CD3, CD4, CD8, F4/80, CD11b, and PD1 by flow cytometry. The results exhibited increased expression of CD4+ T cells, CD8+ T cells, TAMs (F4/80+ CD11b+), and PD-1+ cells in the tumor tissue (Supplementary Figure S2). Through immunohistochemistry, we revealed that the expression of CD3, CD20, CD11b, and PD-1 was significantly higher in melanoma patients (Supplementary Figure S4).

In order to better clarify the relationship between lncRNA and TILs or TMB/IPS, we also constructed ceRNA network in the Supplementary Figure S5 and performed KEGG enrichment analysis on the mRNA downstream of lncRNA.

### 3.4. Immune Checkpoints Expression and Response of Patients to ICIs

As immune checkpoint inhibitors (ICIs) target the dysfunctional immune system and enhance the ability of CD8+ T cells in killing tumor cells, the use of immune checkpoint inhibitors transforms the therapeutic outcome of advanced melanoma.

**Figure 6:**
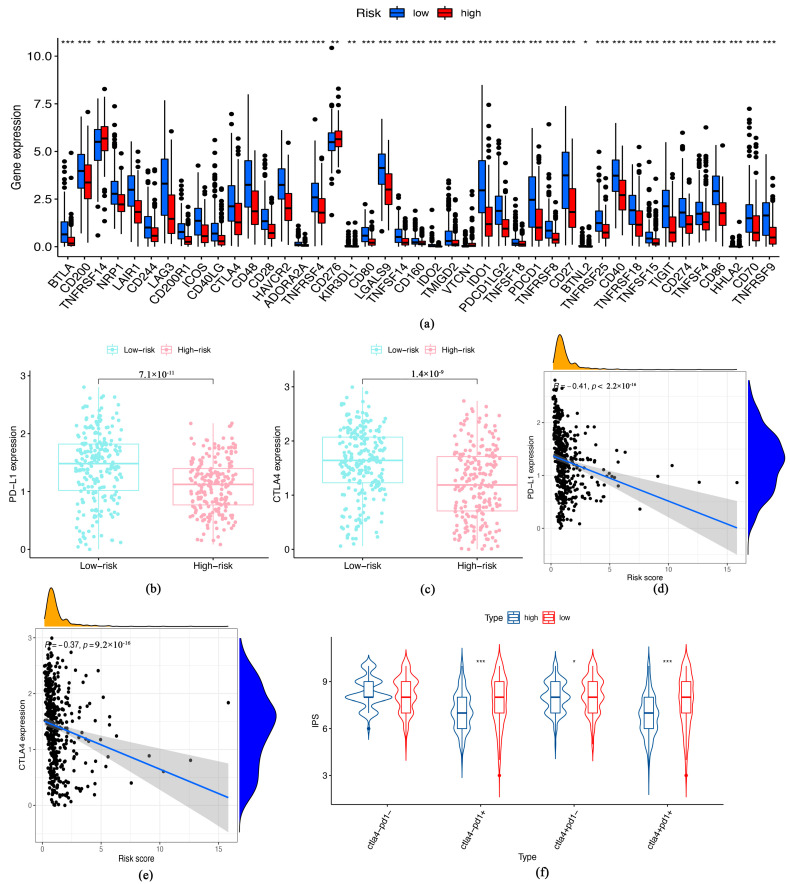
Exploring immune checkpoints and patient response to ICIs. (**a**) The difference in the expression of immune checkpoint-related genes between the high and low-risk groups were examined by the Wilcoxon test. (**b**,**c**) Differences in PD-L1 (**b**) and CTLA-4 (**c**) expression levels in different risk groups. (**d**,**e**) Analysis of the correlation between risk score and PD-L1 (**d**) and CTLA-4 (**e**). (**f**) The score of IPS with PD-L1, CTLA4, CTLA4, and PD1 blockers. p values were shown as: * *p* < 0.05; ** *p* < 0.01; *** *p* < 0.001.

According to our analysis of the TCGA cohort, low-risk patients showed significantly higher levels of immune checkpoint-related genes (Figure 6a). Further, since PD-L1 and CTLA-4 have an important position in immune checkpoints and anti-PDL1 and anti-CTLA-4 inhibitors are widely used in clinical treatment, we explored the correlation of their expression levels with risk score. Significant differences were observed between different risk groups in the PD-L1 and CTLA-4 expression levels (Figure 6b,c). Meanwhile, there was a significantly negative correlation between PD-L1 and CTLA-4 expression and risk score (Figure 6d,e). Using qRT-PCR, we demonstrated the upregulation of PDL1 and CTLA4 in melanoma (Supplementary Figure S3).

Next, we evaluated the score of IPS with PD1, CTLA-4 blockers, and IPS with CTLA4 and PD1 blockers to assess patients’ response of patients to immune checkpoint inhibitor therapy (Figure 6f).

### 3.5. Tumor Burden Mutation (TMB) and Senescence-Related lncRNA Signature

**Figure 7:**
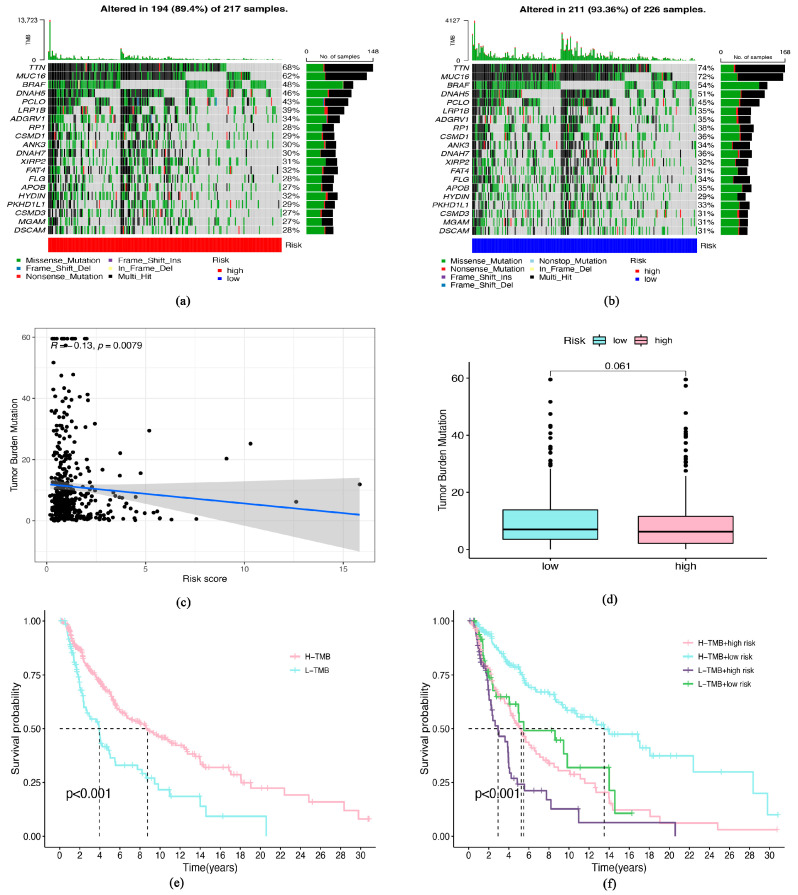
Risk score and tumor burden mutation. (**a**,**b**) The top 20 mutated genes of skin cutaneous melanoma in both risk groups. (**c**) Analysis of the correlation between risk score and TMB. (**d**) Difference between risk score and TMB. (**e**) Kaplan–Meier curves analysis for the differential TMB groups. (**f**) Kaplan–Meier curves for patients clustered by TMB and risk score.

Tumor burden mutation occurs when a gene, or multiple genes, are altered or mutated due to the introduction of a foreign substance, such as a virus, or when there is a mutation of the gene itself. Tumor burden mutation has a significant role to play in the development and progression of cancer. In addition to this, tumor burden mutation can also affect the response of the immune system to the tumor, as well as influence the effectiveness of cancer therapy [24]. We next evaluated somatic mutations in different risk subgroups and identified the top 20 genes mutated most frequently in both risk subgroups. It was noted that TTN, BRAF, MUC16, and DNAH5 mutation rates were greater than 45% in both risk subgroups (Figure 7a,b). As shown in Figure 7c, there is a correlation between risk score and TMB level. Despite not having a significant difference in TMB between both risk groups, patients with lower TMB had a significantly shorter survival time (Figure 7d). The patients with low TMB in the high-risk group also had a worse prognosis, showing a synergistic relationship between the two indicators (Figure 7e,f).

### 3.6. Relationship between the Therapeutic and Senescence-Related Risk Signature

**Figure 8:**
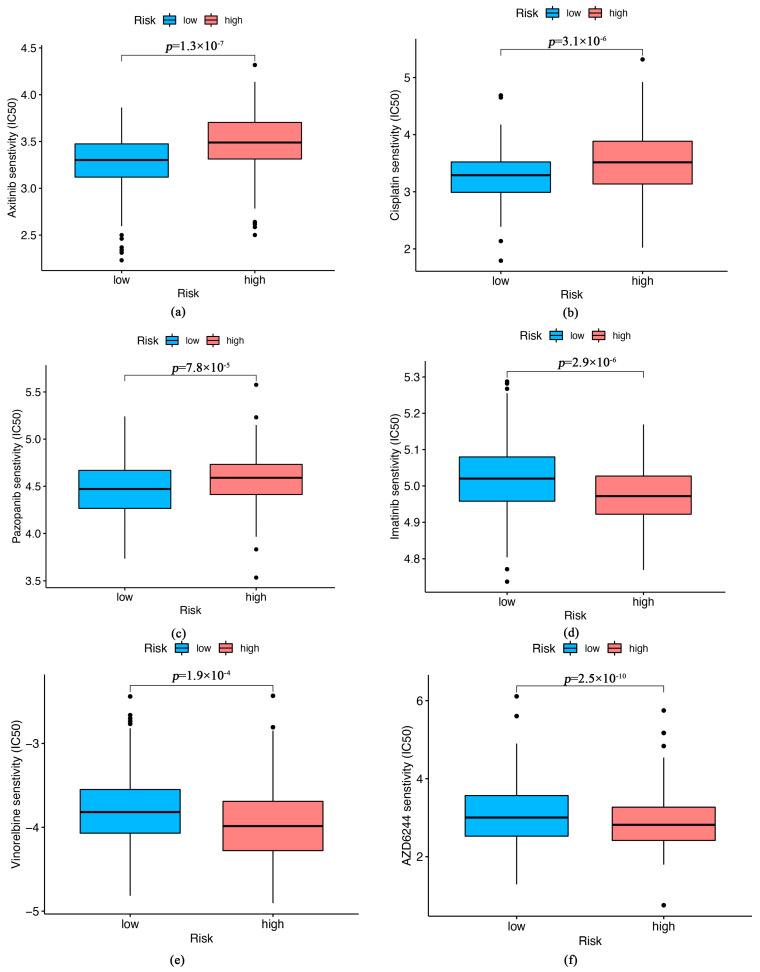
Chemotherapy drug sensitivity analysis. (**a**–**c**) The IC50 of Gefitinib, Mitomycin, and Parthenolide in patients with both risk groups. (**d**–**f**) The IC50 of Docetaxel, Sorafenib, and Imatinib in patients with both risk groups.

For the purpose of guiding the selection of chemotherapy drugs for SKCM patients, a targeted chemotherapy drug sensitivity analysis will be conducted. IC50 values for Axitinib, Cisplatin, and Pazopanib were lower in low-risk patients (Figure 8a–c), suggesting that patients in this risk group had better treatment outcomes with these drugs, while Vinorelbine, AZD6244, and Imatinib exhibited greater sensitivity in the high-risk group (Figure 8d–f).

## 4. Discussion

SKCM is a highly aggressive and lethal tumor. Current treatment mostly relies on surgical treatment and immunotherapy. Induced senescence (also known as quasi-senescence therapy) is currently being extensively researched as a potential cancer treatment. Cellular senescence is considered a favorable mechanism against tumor progression [25]. Currently, it is believed that senescent cancer cells may exert their anticancer effects through a complex interplay between senescence-associated secretory phenotypes (SASP) and immune microenvironments [26]. Nevertheless, no study has yet reported the relevance of cellular senescence-related lncRNAs in evaluating clinical outcomes in immune infiltration in SKCM. Therefore, for the purpose of enhancing the accuracy of predicting patients’ prognosis in SKCM patients and to guide personalized treatment, a novel prognostic signature was developed on the basis of lncRNAs associated with senescence.

In our study, a systematic analysis of senescence-related lncRNAs in SKCM was carried out. One hundred and seventy-nine differently expressed and prognosis-related senescence lncRNAs were identified between SKCM and normal samples. After that, through Lasso-Cox regression analysis, a risk signature was constructed in our training cohort. It contains a total of four senescence-related lncRNAs: AC009495.2, U62317.1, AATBC, and MIR205HG.

Previous studies have partially elucidated the important role played by signature lncRNAs in tumor development and progression. A prognostic model associated with genomic instability was constructed and identified that AATBC promotes invasion of melanoma [27]. In melanoma, the lncRNA MIR205HG promotes tumor growth and invasion via miR-299-3p/VEGFA axis [28]. In addition, MIR205HG promotes esophageal squamous cell carcinoma progression [29].

Several clinically immune-related lncRNAs including AC009495.2 were identified, and a prognostic model was developed, providing a molecular assessment of immune function and a potential prognosis for patients [30]. Jiang et al. developed a risk signature of lncRNA pairs containing lncRNA U62317.1 and MIR4435-2HG for predicting prognosis of breast cancer patients [31].

Next, patients were classified as high and low risk using their median risk score based on senescence-related risk signature. Then, univariate and multivariate Cox regression analyses were employed to demonstrate that the risk score signature was a good independent predictor to evaluate the survival prognosis in SKCM patients. Moreover, we also combined several risk and clinical characteristics to create a nomogram, which allowed us to quantify the survival time of SKCM patients.

GSEA was carried out to analyze the different mechanisms by which senescence-related lncRNAs influence prognosis in SKCM patients. The findings showed enrichment of immune-related pathways in low-risk groups. The role of the tumor microenvironment is to provide a supportive environment for tumor growth and maintenance, which includes the extracellular matrix, blood vessels, and immune cell [32]. The tumor microenvironment has a direct impact on the aggressiveness of melanoma. It can directly control the growth and spread of cancer by activating or suppressing the immune response. In addition, the tumor microenvironment can alter the phenotype of tumor cells by affecting their proliferation, survival, and drug sensitivity [33].

To better guide the immunotherapy of SKCM, we then evaluated the immune infiltration and TME scores in both risk groups to better guide the immunotherapy of SKCM. During the analysis of immune infiltration in different risk groups, a higher expression of activated memory T cells and CD8+ T cells was found in the low-risk group, whereas the expression of M2 macrophages was higher in the high-risk group. These discoveries are consistent with previous findings that cytotoxic CD8+ T cells are able to eliminate pathogens and tumor cells and preserve long-term immune protection in the body [34]. M2 macrophages play a crucial function in tumorigenesis, invasion, and immune evasion [35].

Cancer immunotherapy is widely defined as a treatment that targets any component of the immune system involved in anti-tumor immunity directly or indirectly. Cancer immunotherapy has changed the outlook for cancer therapy, with the increasing use of immune checkpoint inhibitors targeting different molecules and greatly improving the rate of 5-year survival for patients [36]. During our research, we discovered significantly higher levels of immune checkpoints in the low-risk group, predicting the signature we have established might offer certain guidelines for the treatment of ICIs and could help us lay the foundation for establishing a more personalized immunotherapy regimen for SKCM patients. We also found that Axitinib, Cisplatin, and Pazopanib exhibited greater sensitivity in the low-risk group, whereas Vinorelbine, AZD6244, and Imatinib exhibited greater sensitivity in the high-risk group, which could also contribute to the development of better therapeutic measures for the clinical treatment of patients receiving neoadjuvant chemotherapy.

Despite the predictive value of the risk signature we constructed, there are still a few limitations. First, more datasets are required to validate its accuracy. Second, the conclusions derived from bioinformatics analysis need to be verified by more experimental analyses.

## Data Availability

Publicly available datasets were analyzed in this study. This data can be found in the UCSC Xena (http://xena.ucc.edu/, accessed on 20 March 2022) and the Genotype Tissue Expression database (https://gtexportal.org/home/, accessed on 21 March 2022).

## Supplementary Materials

The following supporting information can be downloaded at: https://www.mdpi.com/article/10.3390/biom13040661/s1, Table S1: Characteristics of patients in low- and high-risk score in TGGA cohort; Table S2 Summary of 210 senescence-related genes; Table S3:The sequence of primers are as follows, Figure S1: RT-PCR showing signature lncRNA expression of human melanoma cell line A375 and epithelial cell line HaCaT, Figure S2: Flow Cytometry was used to detect the proportion of PD-1+ cells, TAMs(F4/80+Cd11b+), CD3+CD4+ and CD3+CD8+ T cells in mouse melanoma, Figure S3. RT-PCR showing PDL1 and CTLA4 expression of normal and tumor, Figure S4: The expression of CD3, CD20, PD-1 and CD11b in melanoma was evaluated by immunohistochemistry, Figure S5: Construction of ceRNA network. (a) CeRNA network was constructed by Cytoscape. (b) The KEGG enrichment analysis of mRNA that is downstream of signature lncRNA.
