# Supplementary material for: Senescence-Related lncRNA Signature Predicts Prognosis, Response to Immunotherapy and Chemotherapy in Skin Cutaneous Melanoma
Source: Biomolecules. 2023 Apr 9;13(4):661. doi: 10.3390/biom13040661 (PMC10135470; doi:10.3390/biom13040661)
Supplement: Supplementary file 1 [file biomolecules-13-00661-s001.zip › biomolecules-2088289-supplementary.pdf]

biomolecules

MDPI

Article

Senescence-Related lncRNA Signature Predicts Prognosis, Response to Immunotherapy and Chemotherapy in Skin Cutaneous Melanoma

Kefan Lin [1], Yingtong Zhou [1], Yanling Lin [1], Yuanyuan Feng [1] and Yuting Chen [2] and Longmei Cai [1,*]

[1] Department of Radiation Oncology, Nanfang Hospital, Southern Medical University, Guangzhou, China; linkefan316@outlook.com (K.L.); lystone3@outlook.com (Y.Z.); 1120011070@smu.edu.cn (Y.L.); fyy2333@outlook.com (Y.F.)
[2] Southern Medical University, Guangzhou, China; yutingcc2023@outlook.com
* Correspondence: clm520@i.smu.edu.cn

Supplementary Materials

Table S1. Characteristics of patients in low- and high-risk score in TGGA cohort.

	**High** *N* = *215*	**Low** *N* = *232*
Age	59.1 (15.1)	56.5 (15.9)
Gender		
FEMALE	77 (35.8%)	91 (39.2%)
MALE	138 (64.2%)	141 (60.8%)
T		
T0	5 (2.33%)	18 (7.76%)
T1	14 (6.51%)	27 (11.6%)
T2	35 (16.3%)	41 (17.7%)
T3	42 (19.5%)	47 (20.3%)
T4	89 (41.4%)	55 (23.7%)
Tis	6 (2.79%)	1 (0.43%)
TX	15 (6.98%)	27 (11.6%)
unknow	9 (4.19%)	16 (6.90%)
M		
M0	195 (90.7%)	207 (89.2%)
M1	9 (4.19%)	12 (5.17%)
unknow	11 (5.12%)	13 (5.60%)
N		
N0	112 (52.1%)	110 (47.4%)
N1	36 (16.7%)	37 (15.9%)
N2	19 (8.84%)	30 (12.9%)
N3	25 (11.6%)	29 (12.5%)
NX	18 (8.37%)	14 (6.03%)
unknow	5 (2.33%)	12 (5.17%)
Stage		
I/II NOS	4 (1.86%)	6 (2.59%)
Stage 0	5 (2.33%)	1 (0.43%)
Stage I	27 (12.6%)	49 (21.1%)
Stage II	82 (38.1%)	51 (22.0%)
Stage III	77 (35.8%)	92 (39.7%)
Stage IV	9 (4.19%)	11 (4.74%)
unknow	11 (5.12%)	22 (9.48%)

[a] Median (IQR); *n* (%).

Table S2. Summary of 210 senescence-related genes.

MAP2K7	MRE11	CTC1	CBX4	ID1
WNT1	TNRC6A	MORC3	LMNB1	FBXO5
MNT	ACD	NPM1	NEK4	BGLAP
SPI1	KIR2DL4	PRELP	HRAS	CDC23
PDCD4	TNIK	IGFBP7	RSL1D1	TNRC6C
IGF1R	RB1	UBE2C	FOS	LMNA
CXCL8	NUAK1	ANAPC4	CCNA1	HMGA1
EP400	UBE2E1	TERF2IP	ZMIZ1	CDKN2A
ANAPC1	MAP2K1	MAPK9	YBX1	KDM6B
MAP3K3	AKT3	KAT6A	POT1	CDK6
MAGEA2B	MAPK14	NEK6	ABI3	STAT3
BRCA2	MOV10	PRKCD	PTEN	TBX3
AGO4	UBB	MIR590	TNRC6B	RBBP4
ARNTL	TFDP2	MAPKAPK3	MDM2	PHC3
WNT16	EEF1E1	FOXM1	RBL1	CDKN1B
ZKSCAN3	SIRT1	MAP2K3	ANAPC10	ING2
PHC2	VASH1	MAPKAPK2	CBX8	UBE2S
PML	SMC6	ANAPC7	ERCC1	EHMT2
ASF1A	ANAPC15	TFDP1	IL1A	CGAS
EHMT1	ANAPC5	ATR	CDC27	E2F1
RPS6KA2	MDM4	RELA	ARG2	TP53
TP63	CALR	MAPK1	CDKN2B	TWIST1
PLK2	SOD1	MAP4K4	CDKN2C	MAPK8
SMC5	AGO3	ERF	FZR1	E2F3
SRF	EED	CBX6	ETS2	RPS6KA1
CHEK1	CDC26	NUP62	SCMH1	ID2
RNF2	CCNE1	NBN	UBN1	IL6
KAT5	ROMO1	MAPK11	CEBPB	MAGEA2
OPA1	LIMS1	SUZ12	ETS1	TINF2
CBX2	DNAJA3	TERT	B2M	PAWR
ULK3	NFKB1	ANAPC2	BCL6	PRMT6
CDKN1A	ABL1	WRN	BMI1	RWDD1
TERF2	CDK2	TBX2	ANAPC16	ANAPC11
HIRA	ZMPSTE24	CCNE2	MIF	CITED2
EZH2	HLA-G	AGO1	MAPK7	UBA52
PNPT1	MAPK10	JUN	NSMCE2	NOX4
BMPR1A	CCNA2	RPS6KA3	TXN	RPS27A
CDK4	TERF1	BCL2	ATM	CABIN1
MAP2K6	MAP3K5	VENTX	MAPKAPK5	
MAP2K4	HMGA2	PLA2R1	CDKN2D	
SP1	CDC16	MINK1	MAPK3	
RING1	UBE2D1	YPEL3	PHC1	
UBC	E2F2	CDK1	RBBP7	

Table S3. The sequence of primers are as follows.

	**Forward**	**Reverse**
PD-L1	GCTCCAAAGGACTTGTACGTG	TGATCTGAAGGGCAGCATTTC
CTLA-4	CATGGTGTCGCCAGCTTTC	GGTAATCTAGGAAGCCCACTGTA
AATBC	AAGGCCGGTTATCAACGT	GCCAGTCCCTCACTGCTCT
MIR205HG	ATCTCTCAAGTACCCATCTTGGA	GGCCTCATGGTTGTCAGCTC
U62317.1	CCTCTGTGATCCAGCAGGTG	GACAGGAGTGACAGGTGTGG
AC009495.2	AGAGAACCTTTGCTGTCCCTGTTTG	CATCTTCCTGGAGTCTGCGTTGTTC

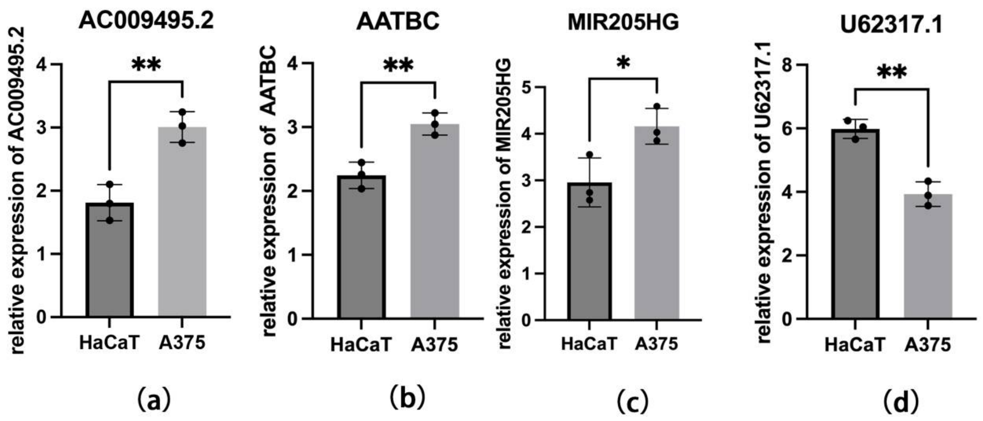

Figure S1. RT-PCR showing signature lncRNA expression of human melanoma cell line A375 and epithelial cell line HaCaT.

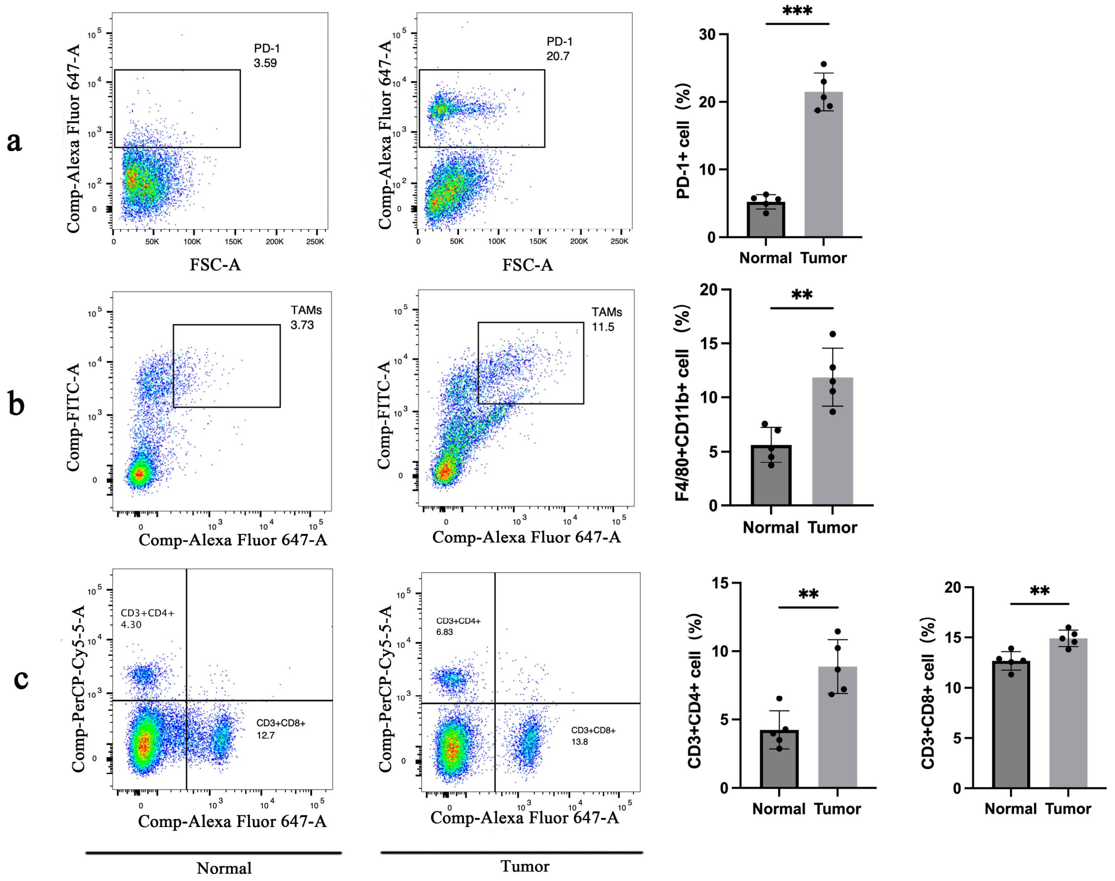

Figure S2. Flow Cytometry was used to detect the proportion of PD-1+ cells, TAMs(F4/80+Cd11b+), CD3+CD4+ and CD3+CD8+ T cells in mouse melanoma. (a) The proportion of PD-1+ cells in the normal (left) and tumor (right). (b) The proportion of CD11b+ cells in the normal (left) and tumor (right). (c) The proportion of CD3+CD4+ and CD3+CD8+ cells in the normal (left) and tumor (right).

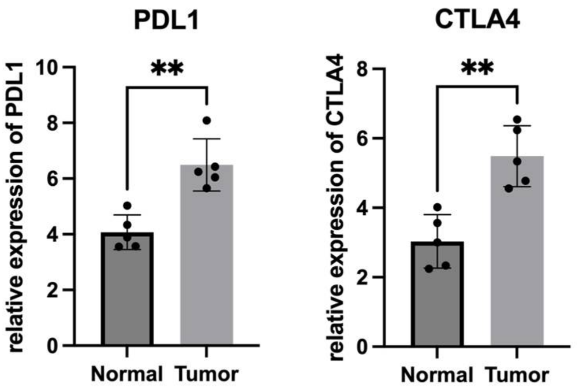

Figure S3. RT-PCR showing PDL1 and CTLA4 expression of normal and tumor.

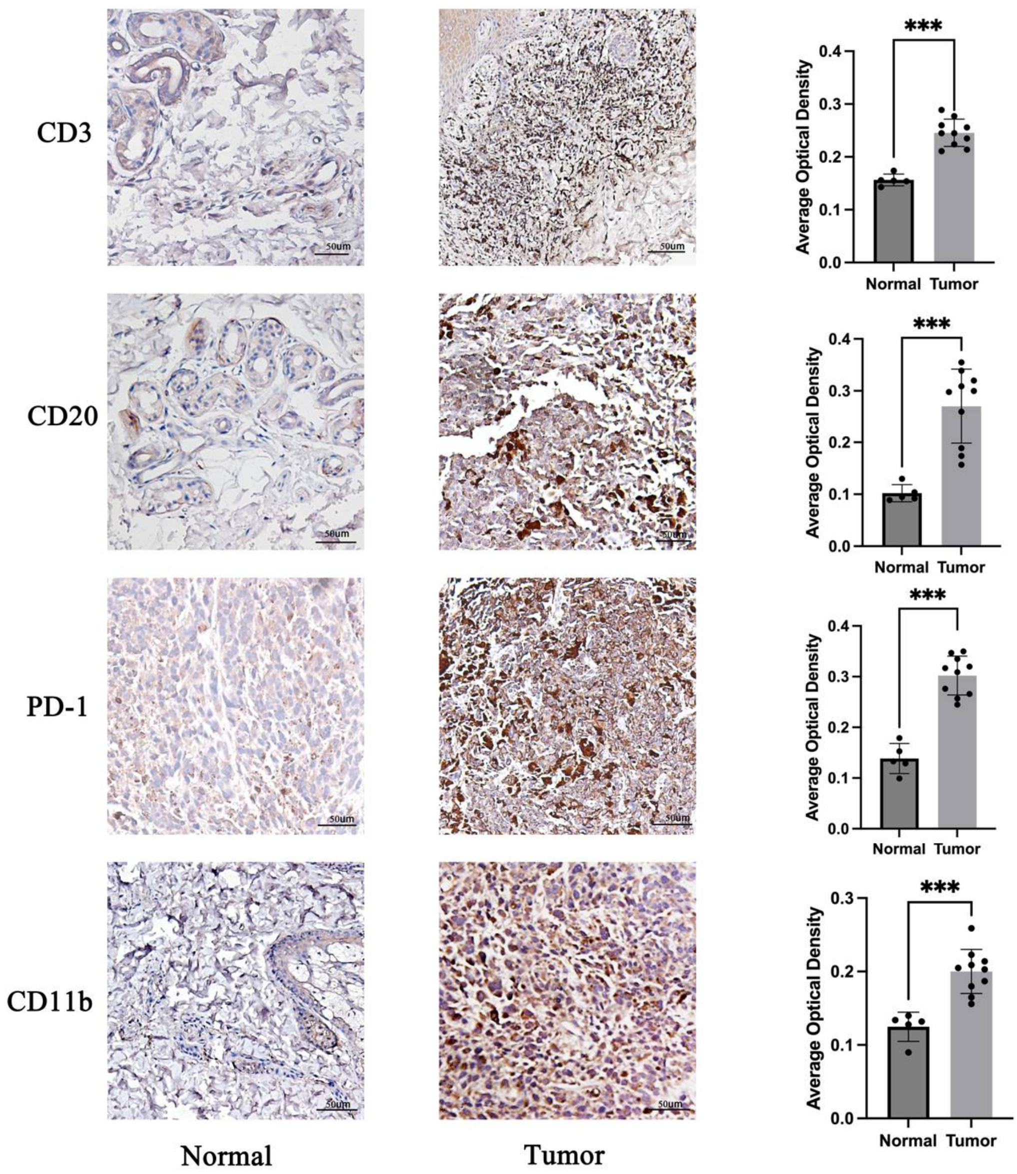

Figure S4. The expression of CD3, CD20, PD-1 and CD11b in melanoma was evaluated by immunohistochemistry.

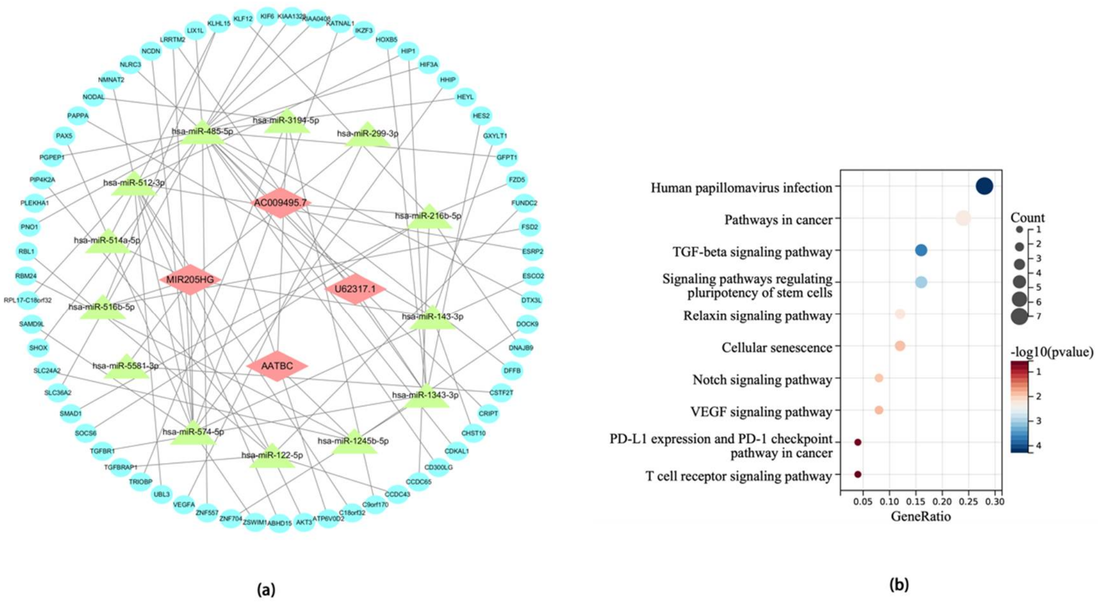

Figure S5. Construction of ceRNA network. (**a**) CeRNA network was constructed by Cytoscape. (**b**) The KEGG enrichment analysis of mRNA that is downstream of signature lncRNA.

Disclaimer/Publisher's Note: The statements, opinions and data contained in all publications are solely those of the individual author(s) and contributor(s) and not of MDPI and/or the editor(s). MDPI and/or the editor(s) disclaim responsibility for any injury to people or property resulting from any ideas, methods, instructions or products referred to in the content.

Copyright: © 2023 by the authors. Submitted for possible open access publication under the terms and conditions of the Creative Commons Attribution (CC BY) license (https://creativecommons.org/licenses/by/4.0/).
